# Concurrent pathogenic variants in *SLC6A1*/*NOTCH1*/*PRIMPOL* genes in a Chinese patient with myoclonic-atonic epilepsy, mild aortic valve stenosis and high myopia

**DOI:** 10.1186/s12881-020-01035-9

**Published:** 2020-05-06

**Authors:** Haiming Yuan, Qingming Wang, Yufeng Li, Shuangxi Cheng, Jianxin Liu, Yanhui Liu

**Affiliations:** 1 Dongguan Maternal and Child Health Care Hospital, Dongguan, 523120 China; 2 Dongguan Institute of Reproductive and Genetic Research, Dongguan, 523120 China

**Keywords:** *SLC6A1*, *NOTCH1*, *PRIMPOL*, Myoclonic-atonic epilepsy, High myopia, Whole-exome sequencing

## Abstract

**Background:**

Pathogenic *SLC6A1* variants have been reported in patients with myoclonic-atonic epilepsy (MAE). *NOTCH1*, encoding a member of the Notch family of proteins, is known to be associated with aortic valve disease. The *PRIMPOL* variant has only been identified in Chinese patients with high myopia. Exome sequencing analysis now allows the simultaneous detection of multiple genetic etiologies for patients with complicated clinical features. However, the presence of three *Mendelian* disorders in one patient supported by their respective pathogenic variants and clinical phenotypes is very rare.

**Case presentation:**

Here, we report a 4-year-old Chinese boy who presented with MAE, delayed language, borderline intellectual disability (ID), mildly impaired social skills and attention deficit hyperactivity disorder (ADHD). He also had mild aortic valve stenosis and high myopia. Using whole-exome sequencing (WES), we identified three variants: (1) *SLC6A1*, NM_003042.4: c.881-883del (p.Phe294del), (2) *NOTCH1*, NM_017617.5:c.1100-2A > G and (3) *PRIMPOL*, NM_152683.4:c.265 T > G (p.Tyr89Asp). Parental Sanger sequencing confirmed that *SLC6A1* and *NOTCH1* variants were de novo, whereas the *PRIMPOL* variant was inherited from the father who also had high myopia. Furthermore, the *PRIMPOL* variant was absent from the genomes of the paternal grandparents, and thus was also a de novo event in the family. All three variants are classified as pathogenic.

**Conclusion:**

The *SLC6A1* variant could explain the features of MAE, delayed language, borderline ID, impaired social skills and ADHD in this patient, whereas the features of aortic valve stenosis and high myopia of the patient may be explained by variants in *NOTCH1 and PRIMPOL*, respectively. This case demonstrated the utility of exome sequencing in uncovering the multiple pathogenic variants in a patient with complicated phenotypes due to the blending of three *Mendelian* disorders.

## Background

*SLC6A1* (MIM 137165) encodes a gamma-aminobutyric acid (GABA) transporter known to be crucial for the reuptake of GABA from the synaptic cleft [1]. GABA is the main inhibitory neurotransmitter that counterbalances neuronal excitation in the brain, and disruption of this inhibitory balance can cause seizures. Previous reports have showed that pathogenic *SLC6A1* variants are associated with MAE in patients by a loss-of-function mechanism in which *SLC6A1* variants reduce or abolish the function of the GAT-1 GABA transporter [2–7]. In addition, patients with MAE may also present with variable degrees of intellectual disability, language impairment and behavioral problems, such as aggressive behavior/irritability, ADHD and autistic features [2, 4–6, 8].

*NOTCH1* (MIM 190198) encodes a member of the Notch protein family, which includes *NOTCH1*, *NOTCH2*, *NOTCH3* and *NOTCH4* receptors [9]. Notch proteins belong to single-pass transmembrane receptors that regulate cell fate determinations during development, ensure crosstalk between different types of cells and their physiological differentiation, and are particularly critical in the development of cardiac valvulogenesis. Evidence suggests that pathogenic variants in this gene are related to aortic valve disease [10–14].

*PRIMPOL* (MIM 615421) encodes a primase-polymerase protein that is ubiquitous expressed in the eye and 26 other tissues [15]. Zhao et al. (2013) identified a heterozygous missense variant (c.265 T > G, p.Y89D) in *PRIMPOL* in affected members of a 4-generation Chinese family with high myopia and in 4 sporadic Chinese patients with myopia. The *PRIMPOL* variant (p.Y89D) segregated with the disease and was absent in 270 Chinese controls, which implicated it in high myopia [16].

Here, we report three concurrent pathogenic variants in *SLC6A1*/*NOTCH1*/*PRIMPOL* genes in a Chinese boy with complicated clinical phenotypes, including MAE, delayed language, borderline ID, behavioral problems, aortic valve stenosis and high myopia.

## Case presentation

The proband, a 4-year-old Chinese boy, was the first-born to a nonconsanguineous couple and had a healthy younger brother. He was born at 38 weeks’ gestation with a weight, height, and head circumference well within the normal ranges. His family history was unremarkable. He sat independently at 6 months, spoke his first word at 7 months, and walked without assistance at 13 months. At the age of 2 years 6 months, his language development showed a gradual delay. Seizures occurred at 2 years 8 months of age, characterized by initial cessation of activity and then progression to a fall sometimes followed by myoclonic movements. The event lasted for 10 s each time with spontaneous remission. The patient was conscious throughout the seizures. Seizures were induced by fatigue, strong light and overexcitement. Seizure types observed included absences and myoclonic-astatic, absence and partial seizures. Brain magnetic resonance imaging was normal at this age, but the electroencephalogram (EEG) was obviously abnormal due to paroxysms of bilateral independent and generalized 2.0–3.0 Hz frontally dominant high-voltage rhythmic spikes/polyspikes-and-slow waves. After valproate therapy was initiated, his myoclonic seizures were controlled to an average of one episode per 2 weeks. He displayed borderline ID and mildly impaired language and had behavioral problems, including obvious ADHD, irritability, mildly impaired social reciprocity and poor eye contact.

Then, he was referred for a comprehensive clinical examination. Mild aortic valve stenosis was revealed by echocardiography. High myopia was also diagnosed, which was probably an inherited event because his father was also affected by high myopia during his early childhood without other suspected ocular diseases. However, his paternal grandparents had totally normal vision.

### Whole-exome sequencing (WES)

This study was approved by the Committee on Ethics of the Dongguan Maternal and Child Health Care Hospital. DNA of family members was extracted from peripheral blood lymphocytes using standard methods and the sample from the proband was sent for whole exome sequencing. The parental DNA was used to confirm suspected variants by Sanger sequencing. WES was operated with an Illumina HiSeq 2500 system (Illumina). The bcl2fastq2 Conversion Software (v2.20) was employed for extracting Fastq files. BWA (v0.2.10) was used for genome alignments and variant detection. Clinic Sequence Analyzer (CSA) software was employed for biological analysis and interpretation. The pathogenicity of the sequence variants was interpreted in accordance with the American College of Medical Genetics and Genomics/Association for Molecular Pathology (ACMG/AMP) guidelines [17].

**Fig. 1:**
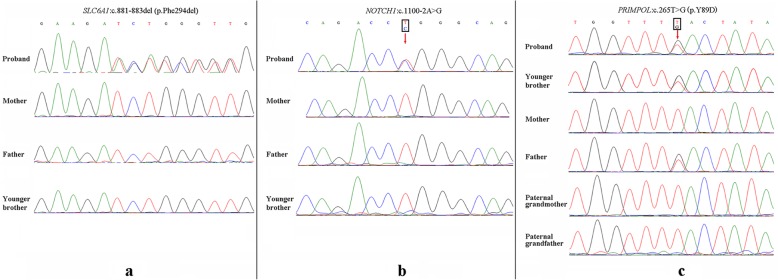
WES identified three concurrent pathogenic variants in our patient. **a** A de novo variant (c.881-883del, p.Phe294del) was identified in *SLC6A1*. **b** A de novo variant (c.1100-2A > G) was identified in *NOTCH1*. **c** A pathogenic variant (c.265 T > G, p. Tyr89Asp) in *PRIMPOL* was identified in our patient and his younger brother and was inherited from the affected father. Furthermore, the variant was absent from the genomes of the paternal grandparents and thus was also a de novo change in the family. Red arrows, mutant bases

Using WES, a de novo inframe deletion variant at chr3:11067490–11,067,492 (hg19) in *SLC6A1* was identified in our patient and was not observed in his parents or younger brother. The *SLC6A1* change, c.881-883del (p.Phe294del), is predicted to cause the deletion of Phe (Fig. 1a). The Phe294 residue is highly conserved among different species. This inframe deletion variant is not present in either the 1000 genome databases or Exome Aggregation Consortium. Furthermore, this variant was previously reported to be a de novo event in patient 13, who was affected by seizures, described by Johannesen et al. (2018) [4]. Thus, this variant was categorized as clinically likely pathogenic according to ACMG/AMP guidelines (PS2 + PM1 + PM2) (PS: pathogenic strong; PM: pathogenic moderate).

**Fig. 2:**
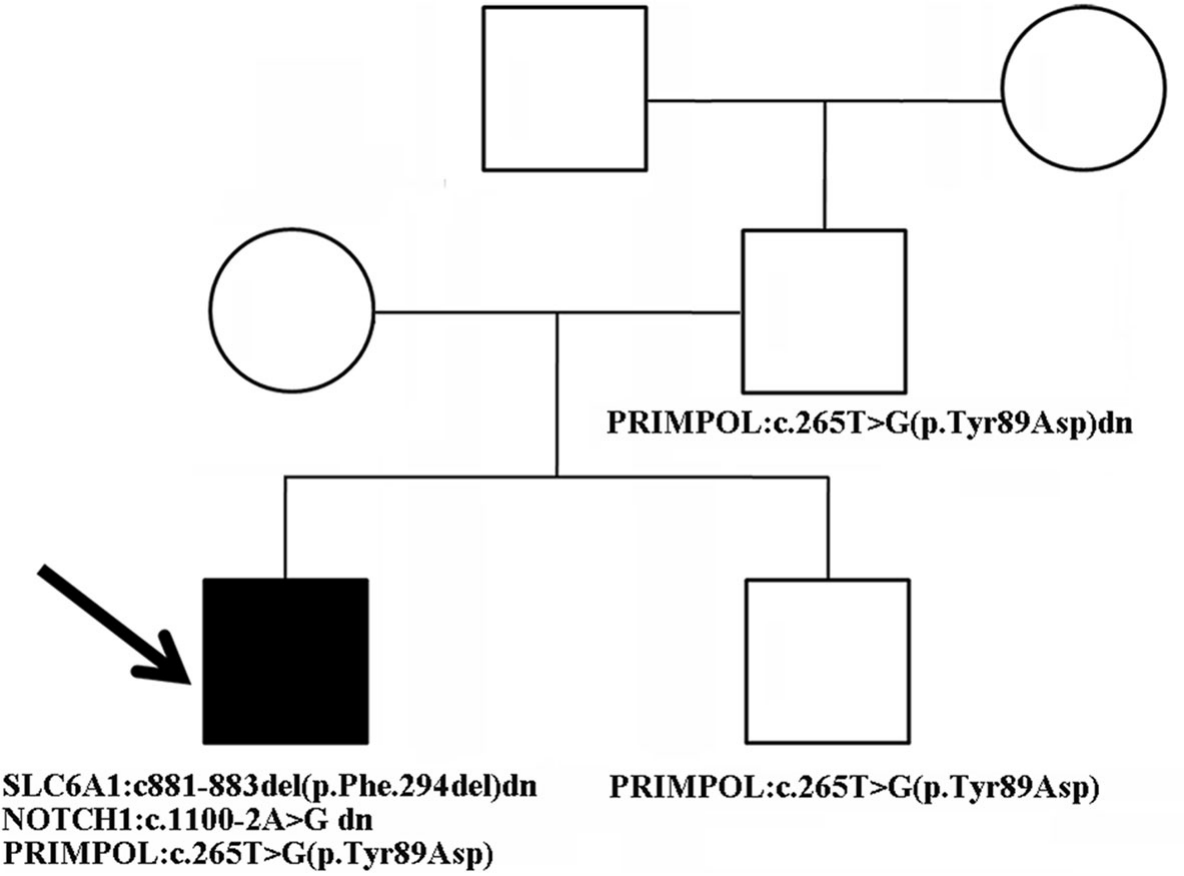
Pedigree of our proband with the variants in *SLC6A1*, *NOTCH1* and *PRIMPOL*. The two variants in *SLC6A1* and *NOTCH1* were de novo events, and the *PRIMPOL* variant was inherited from the affected father, which was also a de novo change since the variant was absent from the paternal grandparents. Proband is marked with an arrow. dn: de novo

Then, the patient was given a comprehensive clinical examination. Unexpectedly, mild aortic valve stenosis and high myopia were revealed. Thus, WES data were reanalyzed to search for potential pathogenic variants causally linked with these clinical features. A de novo variant (c.1100-2A > G) in the *NOTCH1* gene was identified in our patient, and his younger brother did not carry the variant (Fig. 1b). The variant alters the canonical splice acceptor site of intron 6. In silico analysis using Human Splicing Finder v3.0 (Aix Marseille University and Inserm, Marseille, France) predicted that this variant would eliminate the acceptor site, which was speculated to cause exon 7 to be incorrectly spliced out of the RNA transcript. Thus, this variant was classified as clinically pathogenic (PVS1 + PS2) (PVS1: pathogenic very strong). Finally, the variant (c.265 T > G, p.Tyr89Asp) in *PRIMPOL* was identified in the proband and his younger brother, who had not undergone an ophthalmic evaluation for his age, and was inherited from the affected father with high myopia. Further segregation analysis was performed and showed that the variant arose de novo in the father and was not seen in the paternal grandparents (Fig. 1c), moreover, the paternal grandparents had totally normal vision. Furthermore, functional studies have been established to support a damaging effect of the variant on PRIMPOL [18]. According to the ACMG/AMP guidelines, the variant was classified as pathogenic (PS2 + PS3 + PP1). The pedigree of our patient with three *Mendelian* disorders is depicted in Fig. 2.

## Discussion and conclusions

Exome sequencing has been recommended as the first-tier clinical diagnostic test for individuals with neurodevelopmental disorders and provides opportunities for gaining insights into the relationships between specific multilocus genomic variations and disorders [19, 20]. However, the presence of three *Mendelian* disorders in one patient supported by their respective pathogenic variants and clinical phenotypes is very rare.

In this study, we reported a 4-year-old Chinese boy who presented with MAE, borderline ID, delayed language, behavioral problems, mild aortic valve stenosis and high myopia. The three concurrent pathogenic variants were identified in our patient and were responsible for the complicated clinical phenotypes. Initially, the patient was referred to the clinic for MAE as the primary symptom at 2 years and 8 months of age. WES was performed for the patient and identified a de novo inframe deletion variant (c.881-883del, p.Phe294del) in *SLC6A1* that has been reported in patients with MAE, but few *SLC6A1* variant cases have been reported in Chinese patients. Previous reports indicated that individuals with MAE responded effectively to a ketogenic diet or valproic acid [2, 4, 5]. Thus, valproate therapy was recommended and greatly reduced his absence seizures to an average of one episode per 2 weeks. Individuals with MAE commonly display intellectual disability, language impairment and behavioral problems, including aggressive behavior/irritability, ADHD and autistic features [2, 4–6, 8]. Then, neurodevelopmental assessments were performed for the patient. As a result, he was diagnosed with borderline ID, mild delayed language, and behavioral problems, including obvious ADHD, irritability, mild impaired social reciprocity and poor eye contact. The severity of the clinical presentation may differ among patients carrying *SLC6A1* variants, even between patients with the same mutation [2, 4]. Our patient carried the same mutation as patient 13 (Johannesen et al., 2018) [4]. Our patient displayed borderline ID, whereas patient 13 had mild ID before epilepsy onset and moderate ID after epilepsy. Our patient had irritability, whereas patient 13 did not show this feature, but both presented with ADHD. Patient 13 had mild ataxia, whereas our patient did not. Patient 13 became seizure-free with the combined treatment of lamotrigine and ethosuximide, whereas our patient nearly became seizure-free with valproic acid being the most effective drug. Thus, both the severity and prognosis of our patient’s clinical manifestations were better than those of the patient 13. However, cognitive competence and language development should be persistently followed for our patient.

Mild aortic valve stenosis and high myopia as incidental clinical features were revealed for our patient through a comprehensive clinical exam. Then, WES data reanalysis was initiated to search for probable pathogenic variants causally linked with these clinical phenotypes. In return, a de novo variant (c.1100-2A > G) in *NOTCH1* and a pathogenic variant (c.265 T > G, p.Tyr89Asp) in *PRIMPOL* inherited from his father with high myopia were identified, which were missed in the initial data analysis. The main reasons for the initial negative result were due to incomplete recognition of the patient’s phenotypes. Therefore, the improvement in access to more detailed phenotypic data of the patients and the possibility of estimating the changing phenotype over time would contribute to the increased diagnostic yield [21].

Echocardiography revealed mild aortic valve stenosis, which may be a consequence of the de novo variant (c.1100-2A > G) in *NOTCH1* [10–14]. At present, mild aortic valve stenosis does not have an impact on his daily life, but it needs to be regularly examined.

The variant (c.265 T > G, p.Tyr89Asp) in *PRIMPOL* was originally identified in affected members of a 4-generation Chinese family with high myopia and in 4 sporadic Chinese patients with myopia. The evidence that the variant segregated with disease and was absent in 270 Chinese controls indicated that the variant (p.Y89D) in *PRIMPOL* was associated with high myopia [16]. Furthermore, Keen et al. (2014) performed a functional study for the variant, unequivocally establishing that this variant has a significant impact on the function of this enzyme [18]. However, Li and Zhang (2015) provided contradictory evidence that the variant was identified in two of 407 patients with high myopia, 13 of 813 patients with other ocular diseases, and seven of 384 normal controls [22]. Thus, further work is required to establish the relationship between *PRIMPOL* and high myopia [22, 23]. There is no doubt that myopia is a complex genetic trait. However, our patient was only a 4-year-old boy with high myopia, who was obviously little affected by environmental factors. Furthermore, WES also excluded other known genetic causes responsible for ocular disorders to the greatest degree possible. Next, we performed further segregation analysis in the members of the family and showed that the variant was inherited from the father who had been affected by high myopia since early childhood without other suspected ocular diseases, which was a de novo event in that it was not present in the paternal grandparents who had totally normal vision. The evidence for cosegregation in our family may support a role for *PRIMPOL* in high myopia.

WES has allowed clinicians to better understand the pathogenesis of patients with complicated clinical features [19–21]. The improvement of clinical phenotypes is very important for the interpretation or reanalysis of WES data, and the incidental findings of WES analysis will also promote the revelation of relevant phenotypes, as both complement each other and ultimately form a closed loop for accurate diagnosis of genetic diseases.

## Data Availability

The data used and/or analyzed in the present report was deposited in the NCBI BioProject database. The data is accessible via the accession number: PRJNA627312; or via the links: https://www.ncbi.nlm.nih.gov/bioproject/PRJNA627312

## Abbreviations

MAE: Myoclonic-atonic epilepsy
ID: Intellectual disability
ADHD: Attention deficit hyperactivity disorder
WES: Whole-exome sequencing
GABA: Gamma-aminobutyric acid
